# SARS-CoV-2 BA.1 variant is neutralized by vaccine booster-elicited serum, but evades most convalescent serum and therapeutic antibodies

**DOI:** 10.1126/scitranslmed.abn8543

**Published:** 2022-04-05

**Authors:** Sabrina Lusvarghi, Simon D. Pollett, Sabari Nath Neerukonda, Wei Wang, Richard Wang, Russell Vassell, Nusrat J. Epsi, Anthony C. Fries, Brian K. Agan, David A. Lindholm, Christopher J. Colombo, Rupal Mody, Evan C. Ewers, Tahaniyat Lalani, Anuradha Ganesan, Emilie Goguet, Monique Hollis-Perry, Si’Ana A. Coggins, Mark P. Simons, Leah C. Katzelnick, Gregory Wang, David R. Tribble, Lisa Bentley, Ann E. Eakin, Christopher C. Broder, Karl J. Erlandson, Eric D. Laing, Timothy H. Burgess, Edward Mitre, Carol D. Weiss

**Affiliations:** ^1^ Division of Viral Products, Office of Vaccine Research and Review, Center for Biologics Evaluation and Research, U.S. Food and Drug Administration; Silver Spring, Maryland, USA, 20993.; ^2^ Infectious Disease Clinical Research Program, Department of Preventive Medicine and Biostatistics, Uniformed Services University of the Health Sciences; Bethesda, MD, USA, 20814.; ^3^ Henry M. Jackson Foundation for the Advancement of Military Medicine, Inc.; Bethesda, MD, USA, 20817.; ^4^ U.S. Air Force School of Aerospace Medicine, Wright-Patterson Air Force Base; OH, USA, 45433.; ^5^ Brooke Army Medical Center, Joint Base San Antonio-Fort Sam Houston; TX, USA, 78234.; ^6^ Department of Medicine, Uniformed Services University of the Health Sciences; Bethesda, MD, USA, 20814.; ^7^ Madigan Army Medical Center, Joint Base Lewis McChord; WA, USA, 98431.; ^8^ William Beaumont Army Medical Center, El Paso; TX, USA, 799218; ^9^ Fort Belvoir Community Hospital, Fort Belvoir; VA, USA, 22060.; ^10^ Naval Medical Center Portsmouth, Portsmouth; VA, USA, 23708.; ^11^ Walter Reed National Military Medical Center; Bethesda, MD, USA, 20889.; ^12^ Department of Microbiology and Immunology, Uniformed Services University of the Health Sciences; Bethesda, MD, USA, 20814.; ^13^ Viral Epidemiology and Immunity Unit, Laboratory of Infectious Diseases, National Institute of Allergy and Infectious Diseases, National Institutes of Health; Bethesda, MD, USA, 20892.; ^14^ Clinical Trials Center, Infectious Diseases Directorate, Naval Medical Research Center; Silver Spring, MD, USA, 20910.; ^15^ General Dynamics Information Technology; Falls Church, VA, USA, 22042.; ^16^ Office of the Assistance Secretary for Preparedness and Response, U.S. Department of Human Health and Services; Washington D.C., USA, 20201.; ^17^ Division of Microbiology and Infectious Diseases, National Institute of Allergy and Infectious Disease, National Institutes of Health; Rockville, Maryland, USA, 20892.; ^18^ Influenza and Emerging Infectious Diseases Division, Biomedical Advanced Research and Development Authority, U.S. Department of Health and Human Services; Washington, D.C., USA, 20024.

## Abstract

The rapid spread of the highly contagious Omicron variant of severe acute respiratory syndrome coronavirus 2 (SARS-CoV-2) along with its high number of mutations in the spike gene has raised alarms about the effectiveness of current medical countermeasures. To address this concern, we measured neutralization of the Omicron BA.1 variant pseudovirus by post-vaccination serum samples after two and three immunizations with the Pfizer/BioNTech162b2 SARS-CoV-2 mRNA (Pfizer/BNT162b2) vaccine, convalescent serum samples from unvaccinated individuals infected by different variants, and clinical-stage therapeutic antibodies. We found that titers against the Omicron variant were low or undetectable after two immunizations and in many convalescent serum samples, regardless of the infecting variant. A booster vaccination increased titers more than 30-fold against Omicron to values comparable to those seen against the D614G variant after two immunizations. Neither age nor sex were associated with differences in post-vaccination antibody responses. We also evaluated eighteen clinical-stage therapeutic antibody products and an antibody mimetic protein product obtained directly from the manufacturers. Five monoclonal antibodies, the antibody mimetic protein, three antibody cocktails, and two polyclonal antibody preparations retained measurable neutralization activity against Omicron with a varying degree of potency. Of these, only three retained potencies comparable to the D614G variant. Two therapeutic antibody cocktails in the tested panel that are authorized for emergency use in the United States did not neutralize Omicron. These findings underscore the potential benefit of mRNA vaccine boosters for protection against Omicron and the need for rapid development of antibody therapeutics that maintain potency against emerging variants.

## Teaser

Convalescent serum, serum from vaccinated individuals, and therapeutic antibodies neutralize the SARS-CoV-2 Omicron variant with different potencies.

## INTRODUCTION

In November 2021 a new severe acute respiratory syndrome coronavirus 2 (SARS-CoV-2) variant, named Omicron (Pango lineage B.1.1.529 or BA.1), was identified as a variant of concern. Its rapid spread and unusually high number of mutations, especially in the spike gene, has triggered intense international efforts to track the variant’s spread and evaluate its effects on the potency of therapeutics and vaccines. The Omicron BA.1 variant has 39 amino acid substitutions, including 6 deletions and 3 insertions in the spike protein. Fifteen substitutions are in the receptor binding domain (RBD). The RBD mediates virus attachment to the angiotensin converting enzyme 2 (ACE2) receptor on target cells and is the principal target of neutralizing antibodies that contribute to protection against SARS-CoV-2 infection and coronavirus disease 2019 (COVID-19). Many of these RBD changes have been previously reported to reduce the effectiveness of several therapeutic neutralizing antibodies (reviewed in Corti *et al*. (*1*)). Recent studies indicate substantial immune evasion to two-dose vaccine-elicited serum samples (*2*–*20*), convalescent serum samples (*3*–*14*), and many monoclonal antibodies (*2*–*4*, *12*–*14*, *21*, *22*), though vaccine boosters appear to help overcome immune evasion to some extent (*2*–*10*, *18*). However, study populations and methods vary widely among the studies to date, and many lack critical details about sample timing and host characteristics, including host subclinical exposure history in those vaccinated. Moreover, studies have not examined how host demography predicts neutralizing humoral responses. An examination of how prior infection by a broader diversity of SARS-CoV-2 Delta and non-Delta genotypes is also important for further insights into how genetic diversity may correlate with cross-neutralizing antibody responses.

Here we used lentiviral pseudoviruses to measure antibody neutralization of the SARS-CoV-2 Omicron BA.1 variant in three important contexts: (1) antibodies induced after two and three immunizations with the Pfizer/BNT162b2 vaccine in the same individuals; (2) antibodies induced from infection by different SARS-CoV-2 variants; and (3) eighteen therapeutic antibody products and one antibody mimetic protein product currently in clinical use under an emergency use authorization (EUA) or in late stages of clinical development. We compared the magnitude of neutralization escape by Omicron to the D614G and Delta SARS-CoV-2 variants to help inform public health decisions and to offer further data to understand correlates of protection.

## RESULTS

### Three immunizations with the Pfizer/BNT162b2 mRNA vaccine improved neutralizing antibodies titers to Omicron.

The emergence of Omicron coincided with recommendations for booster immunizations, particularly for at risk populations. We studied the neutralization titers of 39 generally healthy, adult healthcare workers participating in the Prospective Assessment of SARS-CoV-2 Seroconversion study (PASS study, Table 1) (*23*) who received the full primary series (1^st^ and 2^nd^) and booster (3^rd^) immunizations with the Pfizer/BNT162b2 vaccine. We chose to study serum at peak responses after the full two-dose primary series vaccination rather than after 6 months because titers after 6 months are often very low (*2*, *10*, *24*).

**Table 1.** Demographic data for participants receiving Pfizer/BNT162b2 initial vaccine series and booster.

	**N (%)**
**Sex**	
Female	25 (64.1)
Male	14 (35.9)
**Race**	
White	26 (66.7%)
Asian	8 (20.5%)
Black	4 (10.3%)
Multiracial	1 (2.6%)
**Occupation**	
Nurse	11 (28.2%)
Physician	11 (28.2%)
Physical/Occupational/Recreational Therapist	9 (23.1%)
Medical Technician	3 (7.7%)
Lab Personnel	3 (7.7%)
Social Worker	1 (2.6%)
Psychologist	1 (2.6%)
**Anti-N seroconversion after vaccination and before boost**	
Positive	17 (43.6%)
Negative	22 (56.4%)
**Age**	
Mean age ± SD (range)	45 ± 11 (26 - 69)
**Time between second vaccine and sample collection**	
Mean days ± SD (range)	30 ± 11 (28 - 34)
**Time between second vaccine and booster dose**	
Mean days ± SD (range)	267 ± 14 (218 - 310)
**Time between booster dose and sample collection**	
Mean days ± SD (range)	43 ± 17 (7 - 93)

We compared the neutralization titers of these serum samples against pseudoviruses bearing spike proteins from the following variants: D614G, Omicron (A67V, del69-70, T95I, del142-144, Y145D, del211, L212I, ins214EPE, G339D, S371L, S373P, S375F, K417N, N440K, G446S, S477N, T478K, E484A, Q493R, G496S, Q498R, N501Y, Y505H, T547K, D614G, H655Y, N679K, P681H, N764K, D796Y, N856K, Q954H, N969K, and L981F), and Delta (T19R, G142D, E156-, F157-, R158G, L452R, T478K, D614G, P681R, and D950N).

**Fig. 1.**
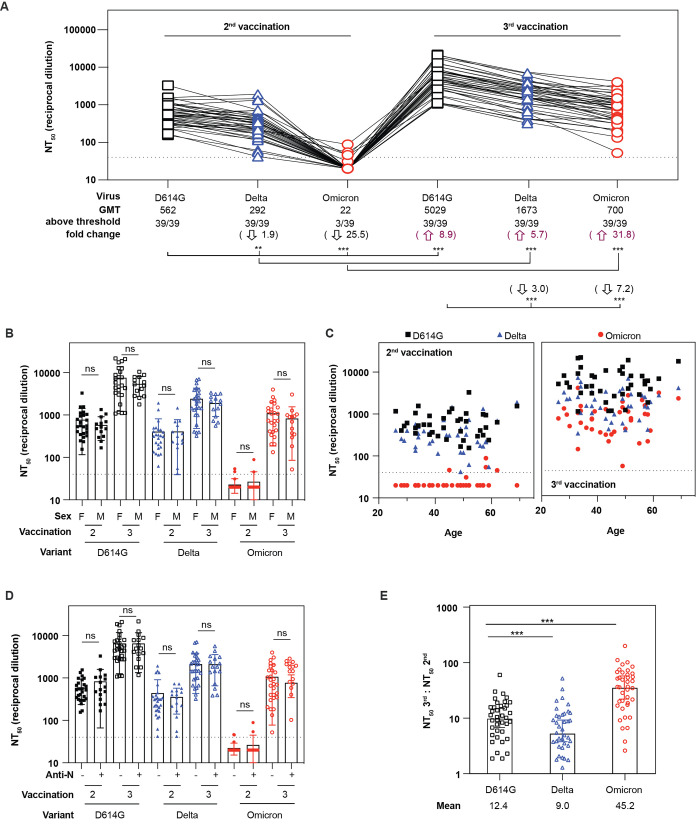
**Sensitivity of the Omicron variant to neutralization by Pfizer/BNT162b2 vaccinee serum samples. (A**) Neutralization assays used lentiviral pseudoviruses bearing SARS-CoV-2 spike proteins from D614G, Delta, or Omicron. Serum samples from 39 healthcare workers were obtained at a mean of 30 ± 11 or 43 ± 17 days after the 2^nd^ or 3^rd^ immunization, respectively. The geometric means titers (GMT), the number of serum samples above threshold (1:40, the lowest dilution tested, dotted lines), and the fold change are indicated. Titers below 1:40 were set at 20 to calculate GMTs. Black arrows indicate fold decrease relative to D614G after the 2^nd^ or 3^rd^ immunization. Purple arrows indicate fold increase for each variant after the 3^rd^ vaccination compared to the 2^nd^ vaccination. Connecting lines indicate serum from the same individual. Demographic information is provided in Table 1. (**B**) NT_50_ values are shown by sex after 2^nd^ or 3^rd^ vaccinations. (**C**) NT_50_ values are shown by age after 2^nd^ and 3^rd^ vaccinations. (**D**) NT_50_ values are shown after 2^nd^ or 3^rd^ vaccination according to anti-N (nucleocapsid protein) seroconversion between 2^nd^ and 3^rd^ vaccinations. Presence of anti-N antibodies suggests prior SARS-CoV-2 infection. (**E**) Ratio of NT_50_ values after 3^rd^ versus 2^nd^ immunizations are shown for D614G, Delta, and Omicron. The mean of ratios is shown below each variant. Black squares correspond to D614G, blue triangles correspond to Delta, and red circles correspond to Omicron. Data shown represent two independent experiments each with an intra-assay duplicate. Significance for (A) and (E) was assessed by one-way ANOVA with the Geisser-Greenhouse correction, followed by Tukey’s multiple comparison test. Significance for (B) and (D) was assessed by Kruskal-Wallis test, followed by Dunn’s multiple comparison test. Significance values are indicated as *P < 0.05; **P < 0.01; ***P < 0.001; ns, nonsignificant.

Only 7.7% (3 of 39) of serum samples obtained at a mean of 30 ± 11 days after the 2^nd^ vaccination had a 50% neutralization titer (NT_50_) against Omicron above our assay threshold (1:40 dilution, Fig. 1A). After the 2^nd^ vaccination, neutralization titers against Omicron (geometric mean titer, GMT=22) were 25.5-fold lower than titers against D614G (GMT=562). By contrast, neutralization titers against Delta (GMT=292) were only modestly lower than D614G. Neutralization titers from the same individuals collected 43 ± 17 days after the 3^rd^ vaccination were 8.9-fold greater against D614G (GMT=5029) than titers after the 2^nd^ vaccination. The titers against Omicron after the 3^rd^ vaccination (GMT=700) were 31.8-fold higher than titers after the 2^nd^ vaccination, whereas titers against Delta after the 3^rd^ vaccination (GMT=1673) were only 5.7-fold higher than titers after the 2^nd^ vaccination. After the 3^rd^ immunization, titers were 3.0- and 7.2-fold lower than D614G for Delta and Omicron, respectively. Importantly, all individuals had measurable neutralizing titers against Omicron after the 3^rd^ vaccination, highlighting the potential for increased protection conferred by a booster vaccine.

We also evaluated whether sex or age might affect titers after vaccinations, but we did not observe a trend for either (Fig. 1B and C). A total of 17 of 39 individuals seroconverted for anti-N (SARS-CoV-2 nucleoprotein) antibodies during regularly scheduled monthly blood sampling between 2^nd^ and 3^rd^ immunizations (*23*, *25*). No symptoms of COVID-19 were reported, possibly indicating silent infection or exposure to SARS-CoV-2. Anti-N seroconversion did not affect neutralizing titers after boosting (Fig. 1D). To assess the breadth of neutralization against Omicron induced by boosting, we compared the change in titers against Omicron or Delta relative to D614G after the 2^nd^ and 3^rd^ vaccinations. To account for variability in the antibody titers among individuals, we calculated the ratio between the neutralization titers after the 3^rd^ and 2^nd^ vaccination for each variant (Fig. 1E). We observed that the mean of the ratios of NT_50_ for the 3^rd^ to 2^nd^ vaccination increased more for Omicron (45.2) than D614G (12.4), whereas the mean of the ratios for Delta was lower (9.0). These findings indicate that the 3^rd^ vaccination increases the response for all three variants and broadened responses to the Omicron variant.

### Neutralization of Omicron by convalescent serum samples from individuals infected by different variants was variable and often low.

**Fig. 2.**
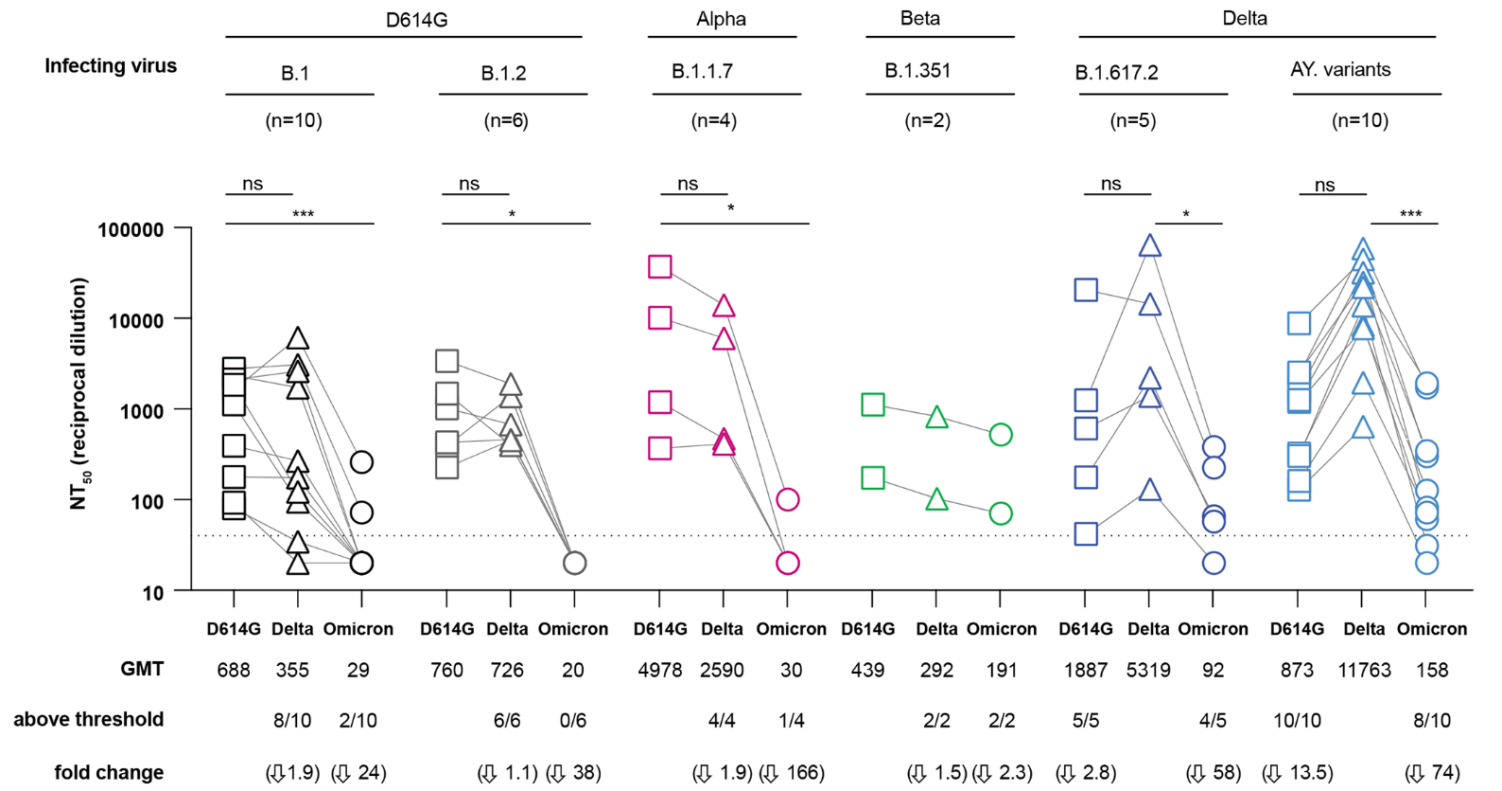
Sensitivity of the Omicron variant to neutralization by convalescent serum samples. Neutralization assays were performed using convalescent serum samples from individuals infected with genotyped variants from B.1, B.1.2, B.1.1.7, B.1.351, B.1.617.2, AY.14, AY.25, AY.44, AY.47, AY.62, AY.74 or AY.119 lineages (Table 2, tables S1 and S2). B.1 and B.1.2 have no mutations in the receptor binding domain and were therefore considered D614G, whereas some of the AY mutants have additional non-RBD spike mutations relative to B.1.617.2. Titers above threshold (1:40) and fold changes are indicated. Titers below threshold were set as 20 for GMT calculations. Arrows indicate decrease relative to D614G (for D614G, Alpha and Beta serum samples) or Delta (for Delta serum samples). Connecting lines indicate serum from the same individual. Data shown represent two independent experiments, each with an intra-assay duplicate. Squares correspond to D614G, triangles correspond to Delta, and circles correspond to Omicron. Significance was assessed using a Kruskal-Wallis test followed by a Dunn’s post-test. Significance values are indicated as *P < 0.05; **P < 0.01; ***P < 0.001; ns, nonsignificant.

We next compared neutralization of D614G (B.1), Delta (B.1.617.2), and Omicron (BA.1) by convalescent serum from unvaccinated individuals that had a prior infection with D614G, Alpha, Beta, or Delta variants (Fig. 2). These individuals were enrolled in the Epidemiology, Immunology, and Clinical Characteristics of Emerging Infectious Diseases with Pandemic Potential (EPICC) study (*26*). Genotypes of the infecting variants were sequenced for all cases (Table 2, tables S1 and S2). These convalescent serum samples were complemented by a Beta convalescent serum sample from another protocol. Neutralization titers for D614G and Alpha convalescent serum samples were significantly reduced against Omicron (B.1, 24-fold, *P* = 0.007; B.1.2; 38-fold, *P* = 0.028; and B.1.1.7, 166-fold, *P* = 0.040) . We note that the convalescent serum samples from individuals infected with Alpha and Beta variants were compared to D614G, rather than the infecting variant. A total of 2 of 10 and 0 of 6 individuals infected with D614G (B.1 and B.1.2) variants, respectively, had responses above threshold against Omicron, whereas 1 of 4 and 2 of 2 individuals infected with Alpha and Beta variants, respectively, were above threshold. Neutralization titers from Delta-convalescent serum were significantly lower against Omicron than against Delta (B.1.617.2, 58-fold, *P* = 0.0133; and AY variants, 74-fold, *P* < 0.0001). Yet, 4 of 5 individuals infected with the B.1.617.2 variant and 8 of 10 individuals infected with AY variants had titers above threshold against Omicron. Additional serum samples from genotyped infections are needed to further investigate whether infection by some variants can induce more cross-neutralizing antibodies to Omicron than other variants.

**Table 2:** Characteristics of unvaccinated individuals providing convalescent serum samples.

	**N = 36**
**Gender**	
Female	13 (36.1%)
Male	23 (63.9%)
**Race**	
White	26 (72.2%)
Asian	1 (2.8%)
Black	6 (16.7%)
Multiracial	3 (8.3%)
**Age**	
Mean age ± SD (range)	39.8 ± 20.2 (1.4 - 73.2)
**Charlson comorbidity index**	
0	19 (52.8%)
1-2	10 (27.8%)
3-4	4 (11.1%)
>5	3 (8.3%)
**Time between infection symptom onset and sample collection**	
Mean days ± SD (range)	30.2 ± 9.3 (14.0 - 51.0)
**Severity of initial infection**	
Outpatient	20 (55.6%)
Hospitalized	16 (44.4%)
Infecting genotype*	
AY.119	1 (2.8%)
AY.14	2 (5.6%)
AY.25	3 (8.3%)
AY.44	1 (2.8%)
AY.47	1 (2.8%)
AY.62	1 (2.8%)
AY.74	1 (2.8%)
B.1	10 (27.8%)
B.1.1.7	4 (11.1%)
B.1.2	6 (16.7%)
B.1.351	1 (2.8%)
B.1.617.2	5 (13.9%)

*Genotypes assigned based on Pango 3.1.17 (2021-12-06). The genotype of the infecting variant was determined in all cases except for one, a traveler who had moderate-severe COVID-19 (outpatient) in the Republic of South Africa during the peak of the Beta (B.1.351) wave in January 2021.

### Boosting reduced apparent antigenic differences between D614G and Omicron variants.

**Fig. 3.**
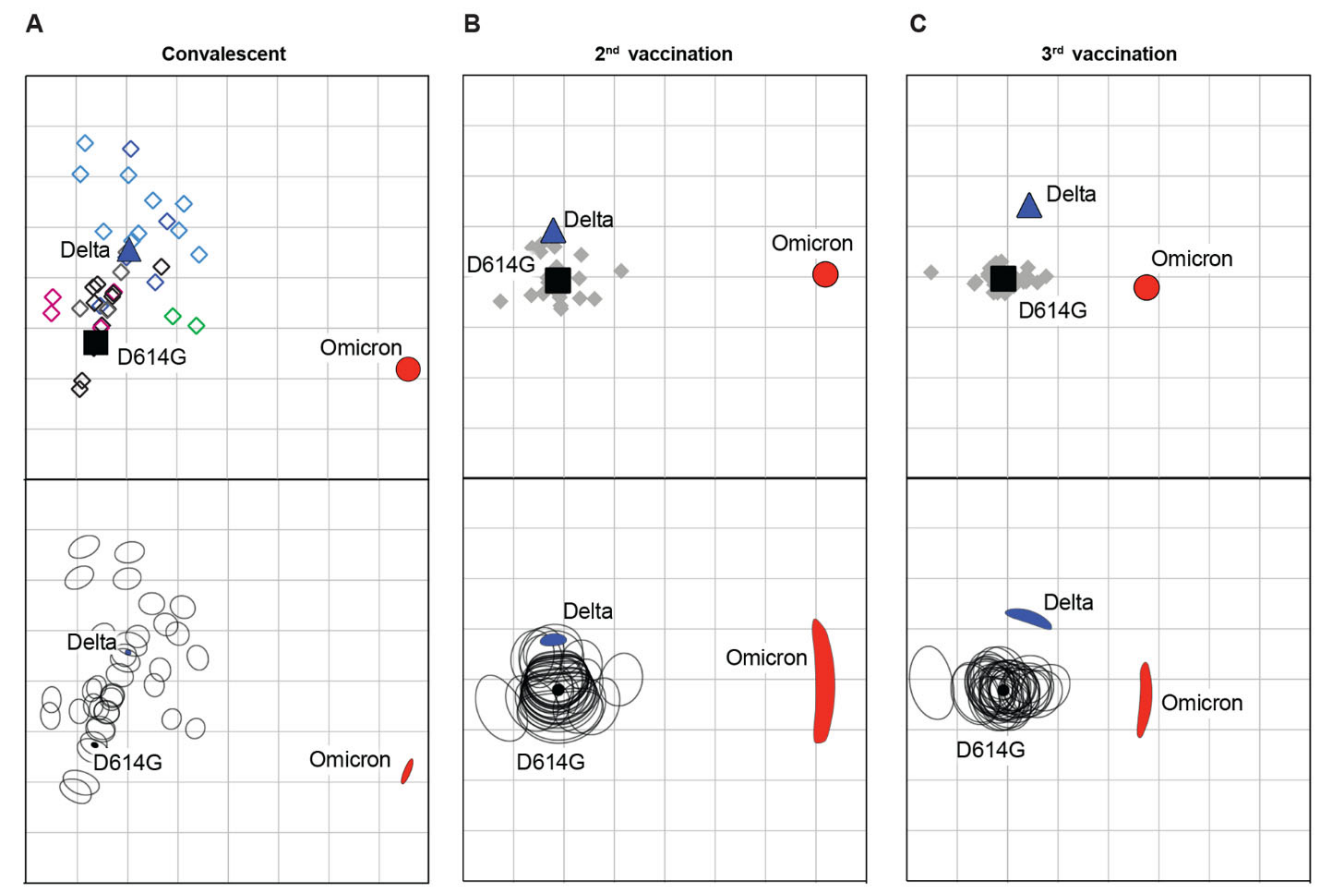
Antigenic cartography of convalescent and vaccinee serum samples against D614G, Delta, and Omicron variants. Antigenic maps were generated from (**A**) convalescent, (**B**) 2^nd^ vaccination, or (**C**) 3^rd^ vaccination serum samples. Convalescent serum samples are shown in open diamonds as follows: B.1 (black), B.1.2 (gray), B.1.1.7 (magenta), B.1.351 (green), AY variants (light blue), and B.1.617.2 (dark blue). Solid gray diamonds correspond to post-vaccination serum. Each grid square (1 antigenic unit) corresponds to a 2-fold dilution in the neutralization assay. Antigenic distance is interpretable in any direction. Black squares correspond to D614G variant. Blue triangles correspond to the Delta variant. Red circles correspond to the Omicron variant. The confidence area of the position of individual viruses or serum sample displayed in the bottom panels was estimated with stress parameter 0.5 and is shown as a rounded shape in the maps.

We applied antigenic cartography to explore how the convalescent and post-vaccination serum samples distinguish the different spike antigens. Antigenic maps were made separately using neutralizing antibody titers from individuals infected with different variants or from individuals after the 2^nd^ or 3^rd^ vaccination (Fig. 3, top panels show antigenic maps, bottom panels show corresponding confidence areas). Convalescent serum samples (Fig. 3A) were more heterogeneous compared to the post-vaccination serum samples (Fig. 3B and C), with D614G and Delta serum samples clustering close to their respective infecting variants, as expected. The 3^rd^ post-vaccination serum samples (Fig. 3C) were more tightly clustered around D614G than the 2^nd^ post-vaccination serum samples (Fig. 3B). In agreement with Fig. 1A and E and Fig. 2, the antigenic distances between Omicron and D614G were large for all three sets of serum.

The antigenic distance between Omicron and D614G was smaller after the 3^rd^ vaccination (7.2-fold difference) than the corresponding antigenic distance for convalescent or post 2^nd^ vaccination serum samples (75.2- and 39.4-fold difference, respectively). Distances between D614G and Delta were small for all three sets of serum. The change in distance between D614G and Delta increased slightly from the 2^nd^ to 3^rd^ immunization, in agreement with Fig. 1E (2.0- and 3.6-fold difference, respectively). Changes in antigenic distances between D614G and variants after the 3^rd^ immunization likely reflect both the increase in titers and the proportion of antibodies specific to D614G or cross-neutralizing to the variants.

### The potency of many therapeutic antibodies is compromised against Omicron.

As part of the United States government COVID-19 response effort to speed development of therapeutics for COVID-19, we assessed the neutralization of Omicron by eighteen therapeutic antibody products and one tri-specific antibody mimetic protein (Designed Ankyrin Repeat Protein, DARPin) currently in clinical use under an EUA or in late stages of clinical development. The therapeutic antibodies included eleven monoclonal neutralizing antibodies (nAbs), five neutralizing antibody cocktails (cnAbs), and two polyclonal antibody preparations. Previously, we reported that several single substitutions in the spike protein of other variants conferred resistance to some of these antibodies (*27*), but a similar assessment has not been performed for the Omicron variant.

**Fig. 4.**
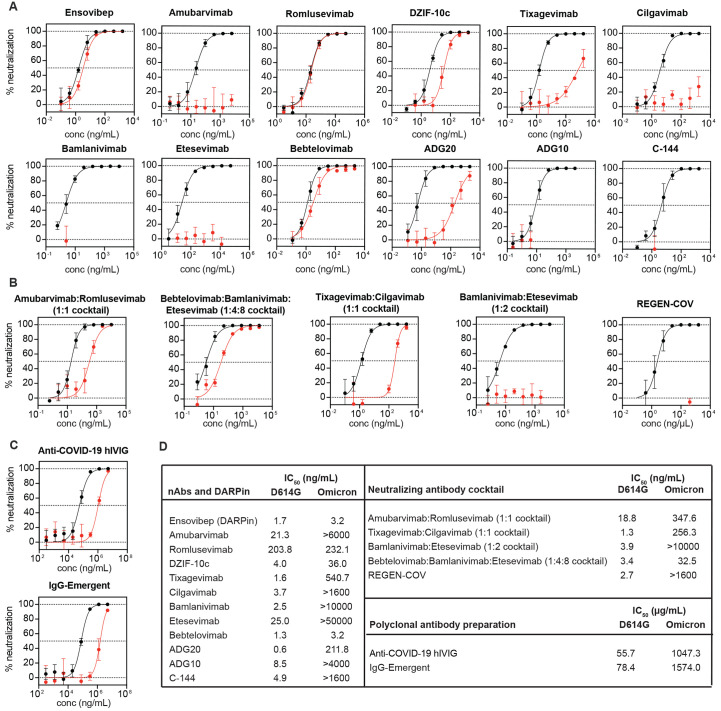
**Neutralization of the Omicron variant pseudovirus by therapeutic antibodies. (A to C)** Neutralization curves against D614G (black) and Omicron (red) are shown for eleven monoclonal neutralizing antibodies (nAb) and one tri-specific antibody mimetic protein (DARPin) (A), five cocktail neutralizing antibody products (B), and two polyclonal antibody preparations (C). (**D**) IC_50_ values against D614G and Omicron are shown for all therapeutic antibodies. IC_50_ values were calculated from two independent experiments, each with an intra-assay duplicate.

Neutralization curves against D614G and Omicron variants were plotted for each therapeutic antibody and the DARPin (Fig. 4A to C). The corresponding 50% inhibitory concentration (IC_50_) values are shown in Fig. 4D. Five nAbs and the DARPin had measurable IC_50_ values against Omicron (3.2-540.7 ng/mL). Romlusevimab (*28*), Bebtelovimab (*29*) and Ensovibep, (DARPin, (*30*)) retained potency comparable to D614G, albeit with varying degrees of absolute potency. DZIF-10c (*31*), Tixagevimab (*32*), and ADG20 (*33*)) retained partial potency to varying degrees. The remaining nAbs (Amubarvimab (*28*), Cilgavimab (*32*), Bamlanivimab (*34*), Etesevimab (*34*), ADG10 (*33*), and C-144 (*35*)) were completely resistant at the highest concentrations tested. Three cnAbs (Amubarvimab:Romlusevimab (*28*), Tixagevimab:Cilgavimab (*36*), and Bebtelovimab:Bamlanivimab:Etesevimab (*29*)) retained partial potency (IC_50_ 32.5-347.6 ng/mL), whereas the remaining cnAbs (Bamlanivimab:Etesevimab (*34*) and REGEN-COV (*37*)) lacked potency at the highest concentrations tested. Many of these findings are consistent with other reports involving similar antibodies (*2*–*4*, *12*–*14*, *21*).

Both polyclonal antibody preparations, Anti-COVID-19 hyperimmune intravenous immunoglobulin (Anti-COVID-19 hIVIG) (*38*) and IgG-Emergent, showed reduced neutralization potency against Omicron (18.8- and 20.1-fold reduction compared to D614G, respectively). These results are consistent with our data from convalescent and vaccinated individuals. Overall, these findings highlight the urgency of continued development of therapeutic antibody products that will remain potent against emerging variants.

## DISCUSSION

Neutralizing antibodies are widely accepted to be an important component of protection against SARS-CoV-2 infection and COVID-19, but efforts to assess antibody titers that correlate with protection are complicated by many factors. These include potential redundancy and synergism of different components of the humoral, cellular, and innate immune system, and differences in variant fitness and host genetics, age, and prior immunity. The risk of infection can also be shaped by human behavior and local public health measures, and measurements of antibody titers can vary with different laboratory methods. Therefore, differences in study populations and laboratory methods are important considerations for assessing the impact of immune evasion by the Omicron variant on medical countermeasures.

Here, using a lentiviral pseudovirus neutralization platform we measured the change in potency of 18 clinical-stage therapeutic antibody products and a DARPin product against Omicron compared to D614G and assessed neutralizing antibodies in serum samples from two well-characterized cohorts of individuals in prospective clinical studies. Our findings show that most vaccinated individuals have low or undetectable titers against Omicron after the second Pfizer/BNT162b2 vaccination, similar to findings reported by others (*2*–*20*). However, the third vaccination significantly increased neutralizing titers (31.8-fold, *P* <0.0001) beyond those elicited by the second vaccination, also in agreement with other recent studies (*2*–*10*, *18*). We note that the post-2^nd^ vaccination and post-3^rd^ vaccination sampling times were similar, indicating that the boost does not simply reflect the time since last vaccine. We found no association between sex or age with neutralizing titers, although the study samples were from generally healthy adults, and the post-vaccine sampling time was short (43 ± 17 days). Continued evaluation of the durability of the boosted titers against Omicron is needed to determine whether titers will decline over time, as has been the case for other variants (*24*). Ultimately, neutralization titers need to be tied to clinical outcomes. Emerging evidence suggests that booster doses substantially lower rates of severe COVID-19 (*39*, *40*).

Recent reports show that infection followed by vaccination results in neutralizing titers comparable or higher than titers achieved after two vaccinations (*2*, *15*, *17*, *18*). The PASS study included anti-N antibody testing on all blood samples for detection of silent infections. The neutralizing antibody titers among the 17 asymptomatic individuals who seroconverted for anti-N antibodies between the 2^nd^ and 3^rd^ immunization were not higher than those who did not seroconvert. We speculate that the lack of additional boosting after N seroconversion may be due to reduced antigen load from incident asymptomatic infection or having reached a maximum response after vaccination.

Antigenic cartography analysis suggests that Omicron is antigenically distant from D614G and Delta variants of SARS-CoV-2, but this distance decreased after the 3^rd^ vaccination. Titers against Omicron were low or non-detectable after the second immunization but boosted in all participants after the 3^rd^ immunization. The apparent broadening of responses to Omicron may be due to the large boost of titers, improved antibody affinity to cross-reactive epitopes, or both. Persistence of germinal center responses and higher degrees of somatic hypermutation with improved antibody affinity has been reported following vaccinations (*41*, *42*). Such affinity maturation may involve increased number of mutations at the antibody-spike protein interface that increase the opportunity for binding (*43*). Recent structural studies of Omicron and other spike proteins reveal complex interactions among multiple residues that may balance receptor interactions with other properties, such as spike protein stability and immune evasion (*11*, *14*, *44*–*46*). Some substitutions specific to the Omicron spike protein near the prefusion-stabilizing 2P mutations (K986P and V987P) used in mRNA vaccines could help stabilize conformations that expose shared epitopes between the D614G vaccine antigen and Omicron.

In contrast to Omicron, the antigenic distance between D614G and Delta increased slightly from the 2^nd^ to 3^rd^ immunization. However, titers against Delta were still higher after the 3^rd^ immunization. Changes in antigenic distances after immunizations may reflect the proportion of D614G-specific antibodies and antibodies that cross-neutralize variants. Cross-neutralizing antibodies elicited by boosting may bind better to some variants than others due to different sets of spike substitutions that affect antibody epitopes or spike protein conformational dynamics. Additional studies with more serum samples and variants are ongoing to investigate how different variants are affected by booster immunizations.

For the convalescent serum samples, titers against Omicron varied widely and were often low. Serum with measurable cross-neutralization titers to Omicron generally had high titers against the infecting variant. Further investigation of potential correlations between neutralization titers against the infecting variant and cross-neutralization titers against other variants are needed.

Most therapeutic antibody products were developed before the emergence of variants of concern. Of the antibody products tested here, only the DARPin and two of eighteen clinical-stage therapeutic antibody products retained potency against Omicron that was comparable to D614G. An additional eight therapeutic antibody products had measurable potency against Omicron to varying degrees. Modest or moderate changes in neutralization potency against a new variant relative to D614G may overcome by high therapeutic concentrations. Importantly, the clinical relevance of changes in IC_50_ has not been determined for any in vitro assay, and the therapeutic concentration of antibodies may be high enough to overcome low degrees of resistance. However, a complete loss of neutralization potency, despite high antibody concentrations, suggests reduced clinical effectiveness. Two cocktail products (Bamlanivimab:Etesevimab and REGEN-COV) that received EUA by the U.S. Food and Drug Administration (FDA) for treatment of COVID-19 lost potency against Omicron. A third EUA product for treatment of COVID-19 (Sotrovimab) has been reported to remain potent against Omicron (*2*, *3*, *12*–*14*, *21*). However, we and others (*27*, *33*) have found that neutralization curves for this antibody plateau at less than 80% neutralization in cells engineered to overexpress ACE2. Because the low plateau precluded us from determining an accurate IC_50_ value, we did not include this antibody in our panel.

Three other cocktail products (Amubarvimab:Romlusevimab, Tixagevimab:Cilgavimab, and Bamlanivimab:Etesevimab:Bebtelovimab) remained potent to varying degrees. Amubarvimab:Romlusevimab and Bamlanivimab:Etesevimab:Bebtelovimab likely retained potency due to one active component. Tixagevimab:Cilgavimab recently received an EUA for prevention of COVID-19 and was more potent than the individual antibody components, implying synergy among the antibodies, as was recently reported (*44*). Combinations of antibodies, especially ones that may synergize, likely provide better resilience to emerging variants.

The two polyclonal preparations, derived from convalescent donors in 2020, also retained measurable potency. The higher absolute IC_50_ of these polyclonal preparations compared to monoclonal antibodies reflects the fraction of the IgG preparation that consists of SARS-CoV-2-specific immunoglobulins. Future lots of polyclonal antibody preparations will be produced from donors with antibodies to SARS-CoV-2 variants as they arise in the donor population.

The limitations of our study include moderate sample sizes, restricted timing of serum collection, lack of long-term follow up, limited number of convalescent serum samples and variants examined, limited ability to assess neutralizing antibodies to some epitopes, and use of pseudovirus as a surrogate for authentic SARS-CoV-2. The strengths of this study include use of serum samples from well characterized cohorts with a broad diversity of genotypes, including within-Delta diversity, comparable sampling times between convalescent and vaccinated individuals, and use of cartography method. An additional strength is the availability of participant demographics and subclinical to clinical antigenic exposure history, which allowed for interpretation of how host characteristics may influence Omicron variant-specific humoral immunity in those with and without vaccinations. Finally, we used authentic therapeutic antibody products and a DARPin obtained directly from manufacturers, rather than corresponding antibodies from other sources.

In summary, our findings indicate that booster immunizations with COVID-19 mRNA vaccines may afford increased protection against Omicron by inducing higher neutralization titers than two immunizations or titers induced by SARS-CoV-2 infection with different variants. Many therapeutic antibodies that were developed early in the pandemic lost potency, but some antibodies in development remain potent to differing degrees. The rapid and unpredictable evolution of SARS-CoV-2 therefore requires continued development and assessments of medical countermeasures.

## MATERIALS AND METHODS

### Study Design

Serum samples used for the neutralization assays were obtained from the PASS and EPICC prospective cohort studies. Sample size calculations and randomization were not performed for the subset used in the neutralization studies. Details of the PASS study protocol, including details of the inclusion/exclusion criteria, have been published (*23*)*.* Inclusion criteria included being generally healthy, ≥ 18 years old, and employed at the Walter Reed National Military Medical Center. Exclusion criteria included history of COVID-19, immunoglobulin G (IgG) seropositivity for SARS-CoV-2 and being severely immunocompromised at time of screening. The study was initiated in August 2020, with rolling enrollment and monthly research clinic visits to obtain serum for longitudinal SARS-CoV-2 antibody testing. The subset of participants selected for this study were those who received two doses of Pfizer/BNT162b2 vaccine by January 26, 2021, had no serological or polymerase chain reaction (PCR) evidence of SARS-CoV-2 infection prior to two doses of vaccine, and had received a third dose of Pfizer/BNTech162b2 vaccine by November 18, 2021. No individual included in this analysis had a clinically-apparent, PCR-confirmed SARS-CoV-2 infection during follow-up.

Participants' serum samples were collected monthly through September of 2021, and then quarterly. For antibody binding studies, serum samples were diluted 1:400 and 1:8000 and then screened for immunoglobulin G (IgG) reactivity with SARS-CoV-2 spike protein and nucleocapsid protein (N) using a multiplex microsphere-based immunoassay as previously described (*24*, *25*) SARS-CoV-2 antigens, a prefusion stabilized spike (S-2P) ectodomain trimer (LakePharma) and the N protein (RayBiotech), were coupled to magnetic microspheres. A master mix of spike and N-coupled microspheres were added to each well of a 96-well microtiter plate and incubated with serum samples diluted in 1X phosphate-buffered saline (PBS). Microtiter plates were incubated at room temperature with agitation (900 rpm) for 45 min, after which, wells were washed three times with PBS-Tween20 (0.05%, PBST). Next, a secondary antibody, cross-absorbed anti-human IgG-biotinylated (Thermo Fisher Scientific), diluted 1:5000 in PBST was added to each well. Again, microtiter plates were incubated with agitation for 45 min and washed three times with PBST. To detect antigen-antibody complexes, streptavidin-phycoerythrin (Bio-Rad, Hercules) was diluted 1:1000 and added to each well. Plates were agitated for 30 min, washed three times with PBST, and resuspended with 100 μL of PBST. Antigen-antibody complexes, and the signal of phycoerythrin, were measured using a Bio-Plex 200 HTF multiplexing system (BioRad). IgG concentrations were reported as a median fluorescence intensity and converted to the World Health Organization (WHO) binding antibody unit (BAU/mL).

The EPICC study is a cohort study of U.S. Military Health System (MHS) beneficiaries that includes those with a history of SARS-CoV-2 infection (*26*)*.* Eligibility criteria for enrollment included those presenting to clinical care with COVID-19-like illness and for SARS-CoV-2 PCR testing. The EPICC study has been enrolling since March 2020. For this sample set, EPICC enrollment occurred at six Military Treatment Facilities (MTFs): Brooke Army Medical Center, Fort Belvoir Community Hospital, Madigan Army Medical Center, Naval Medical Center Portsmouth, Walter Reed National Military Medical Center, and the William Beaumont Army Medical Center.

Study procedures for these participants with SARS-CoV-2 infection included collection of demographic data and completion of a clinical case report form (CRF) to characterize acute COVID-19 illness. Biospecimen collection included serial serum samples for immune response analysis and upper respiratory specimen swabs for virological analysis. For all enrolled individuals, we also abstracted MHS-wide healthcare encounter data from the Military Health System Data Repository (MDR) to determine comorbidities. The Charlson comorbidity index was used to rank the number and seriousness of comorbid disease (*47*). Vaccination status was ascertained by the MDR record in addition to the CRF and questionnaire self-report. For this study sample, we selected individuals with serum samples available at 3 to 6 weeks after symptom onset, with complete or near complete and high coverage spike gene sequence availability, with no prior vaccination, and with a diverse set of genotypes, including the Delta variant and non-Delta variants.

### Diagnosis of SARS-CoV-2 infection and genotyping of infections used for convalescent serum samples

SARS-CoV-2 infection was determined by positive PCR clinical laboratory test performed at the enrolling clinical site, or a follow-up upper respiratory swab collected as part of the EPICC study. The specific PCR assay employed at the MTF varied. The follow-up PCR assay (used for EPICC specimens) was the SARS-CoV-2 (2019-nCoV) CDC qPCR Probe Assay research use only kits (IDT). This assay targets two regions of the SARS-CoV-2 nucleocapsid (N) gene (N1 and N2), with an additional control target to detect the human RNase P gene (RP). We considered a positive SARS-CoV-2 infection as positive based on a cycle threshold value of less than 40 for both N1 and N2 gene targets.

Whole viral genome sequencing was performed on extracted SARS-CoV-2 RNA from PCR positive specimens. A 1200bp amplicon tiling strategy was used (dx.doi.org/10.17504/protocols.io.bwyppfvn). Amplified product was prepared for sequencing using NexteraXT library kits (Illumina Inc.). Libraries were run on the Illumina NextSeq 550 sequencing platform. BBMap v. 38.86 and iVar v. 1.2.2 tools were used for genome assembly. The Pango classification tool (https://cov-lineages.org/) was used for genotype classification (version 3.1.17).

The infecting genotype for one individual (Conv-18, table S1) was determined from a viral sequence derived from an alternative sequencing platform (Illumina MiSeq). Briefly, cDNA synthesis was performed with the Superscript IV first-strand synthesis system (Life Technologies/Invitrogen). Multiplex PCR was performed with the ARTIC v3 primer set, designed to amplify overlapping regions of the Sars-CoV-2 reference genome (MN908947.3). Primer and genomic alignment position information is available here: http://github.com/artic-network/artic-ncov2019/tree/master/primer_schemes/nCoV-2019/V1. PCR products were purified with the MinElute PCR purification kit (QIAGEN). Libraries were prepared with the SMARTer PrepX DNA Library Kit (Takara Bio), using the Apollo library prep system (Takara Bio). The libraries were evaluated for quality using the Agilent 2200 TapeStation (Agilent). After quantification by real-time PCR with the KAPA SYBR FAST qPCR Kit (Roche), libraries were diluted to 10 nM.

### Ethics

The PASS (Protocol IDCRP-126) and EPICC (Protocol IDCRP-085) studies were approved by the Uniformed Services University of the Health Sciences Institutional Review Board (IRB) in compliance with all applicable federal regulations governing the protection of human participants. All PASS and EPICC study participants provided informed consent. The Beta variant convalescent serum sample, obtained from a traveler who had moderate-severe COVID-19 in the Republic of South Africa during the peak of the Beta (B.1.351) wave in January 2021, was obtained with informed consent and covered under the US Food and Drug Administration IRB approved expedited protocol # 2021-CBER-045.

### Therapeutic antibodies and DARPin

Eighteen therapeutic antibody products and a DARPin were generously provided by different companies. Ensovibep (DARPin) was generated by Molecular Partners and in-licensed by Novartis). Amubarvimab (BRII-196), Romlusevimab (BRII-198) and the Amubarvimab:Romlusevimab combination were provided by Brii Biosciences. DZIF-10c was provided by Cologne University Hospital. Bamlanivimab, Etesevimab, Bebtelovimab and two different combinations of these were provided by Eli Lilly. Tixagevimab, Cilgavimab and the Tixagevimab:Cilgavimab combination were provided by AstraZeneca. ADG20 and ADG10 were provided by Adagio Therapeutics Inc. C-144 was provided by Bristol-Myers Squibb. REGEN-COV was provided by Regeneron Pharmaceuticals. Anti-COVID-19 hIVIG was provided by Grifols and IgG-Emergent was provided by Emergent BioSolutions.

### Production of spike pseudoviruses

Plasmids encoding the codon-optimized Omicron spike (SARS-CoV-2 S_B.1.1.529_pVRC8400), luciferase reporter (pHR’CMV-Luc), and HIV gag/pol (pCMVΔR8.2) vector were obtained using Simple Letter of Agreements with the Vaccine Research Center, National Institutes of Health, Bethesda, MD. The codon-optimized D614G and Delta spike expression plasmids were purchased from Genscript. One day prior to transfection a total of 5 × 10^6^ 293T cells were seeded in a 100 mm culture dish containing 10 mL Dulbecco’s Modified Eagle Medium (DMEM) complemented with 10% fetal bovine serum, 100 I.U./ml penicillin, 100 μg/ml streptomycin, 1x MEM non-essential amino acids, 2 mM L-glutamine, and 20 mM HEPES (complete DMEM) and incubated overnight at 37°C in a 5% CO_2_ environment. The next day 4 μg of the pcDNA3.1(+) plasmid encoding Delta spike or 0.5 μg of the VRC8400 plasmid encoding the D614G or Omicron spike, 5 μg of Luciferase reporter plasmid DNA, and 5 μg HIV gag/pol were combined with FuGENE-6 transfection reagent (Promega), according to the manufacturer’s instructions. The DNA-FuGENE-6 reaction mixture was transfected to the 293T cells at 50 to 80% confluency in a 100 mm dish containing 10 mL of complete DMEM and incubated at 37°C in a 5% CO_2_ environment for two days. Supernatant containing pseudoviruses was collected and filtered using a 0.45 μm low protein-binding syringe filter. Aliquoted pseudoviruses were stored at −80°C until further use.

### Neutralization Assay

Neutralization assays were performed as previously described on 293T cells stably expressing angiotensin converting enzyme 2 and transmembrane serine protease 2 (293T-ACE2/TMPRSS2 cells, BEI # NR-55293) (*48*). A total of 3 × 10^4^ 293T-ACE2/TMPRSS2 target cells were seeded in each well in a 96-well flat-bottom culture plate one day prior to neutralization assays and incubated overnight at 37°C in a 5% CO_2_ environment. The next day serum or antibody dilutions were prepared as follows: serum samples from vaccinated individuals and from convalescent individuals was diluted 1:20 in complete DMEM, and three-fold serial dilutions were made (8 dilutions total). For antibody neutralization assays, the 200x antibodies stocks were prepared in complete DMEM and four-fold serial dilutions were made (8 dilutions totals). Virus stocks were diluted in complete DMEM to achieve a 2x concentration with 100,000-500,000 relative light units (RLU)/well. An equal volume of serum or antibody dilution and viruses were combined and incubated for 2 hours at 37°C.

After the incubation, media was aspirated from the target cells, and the serum/virus or antibody/virus mixture was added and incubated at 37°C in a 5% CO_2_ environment for 48 hours. After incubation, the mixture was aspirated, and cells were lysed using luciferase cell culture lysis reagent (Promega). A firefly luciferase assay system (Promega) and a Spectramax-L luminometer (Molecular Devices) were used to quantify the virus infectivity.

The percent neutralization was calculated as 1 minus the ratio between the signal for each serum/pseudovirus or antibody/pseudovirus sample over the signal for virus only control times 100. Neutralization curves were generated by plotting the percentage neutralization as a function of concentration or dilution factor and the data were then fitted using nonlinear dose-response regression curve (GraphPad Prism). The serum dilution or antibody concentration corresponding to 50% neutralization was defined as NT_50_ for serum or IC_50_ for antibodies. Each experiment was performed with an intra-assay duplicate, and all experiments were done at least twice. All individual-level data where n<20 are presented in table S2.

### Antigenic cartography

ACMACS antigenic cartography software (https://acmacs-web.antigenic-cartography.org/) was used to create a geometric interpretation of neutralization titers against the D614G, Delta, and Omicron variants. Antigenic distances are measured in antigenic units (AU). One AU corresponds to a two-fold dilution of the antibody in the neutralization assay (n AU = 2^n^-fold titer drop, for example. 2 AU = 4-fold dilution, 3 AU = 8-fold dilution). Each square in the map indicates one AU. Antigenic distance is measured in any direction of the map. The confidence area of the position of the individual viruses or serum samples was estimated with the stress parameter 0.5.

### Statistical Analysis

Means, standard deviation (SD), geometric mean titers (GMT) with 95% confidence intervals were calculated using GraphPad Prism software. Test for significance was applied as indicated in the legend of each figure. Significance values are indicated as *P < 0.05, **P < 0.01; ***P < 0.001; ns, nonsignificant. Analyses were performed using GraphPad Prism 8.4.3 software.

## Supplementary Materials

### Other Supplementary Material for this manuscript includes the following:

MDAR Reproducibility Checklist
