# Supplementary material for: SARS-CoV-2 BA.1 variant is neutralized by vaccine booster-elicited serum, but evades most convalescent serum and therapeutic antibodies
Source: Sci Transl Med. 2022 Apr 5:eabn8543. doi: 10.1126/scitranslmed.abn8543 (PMC8995032; doi:10.1126/scitranslmed.abn8543)
Supplement: Supplementary file 1 — Tables S1 and Table S2 [file scitranslmed.abn8543_sm.pdf]

Science Translational Medicine
AAAS

# Supplementary Materials for

## SARS-CoV-2 BA.1 variant is neutralized by vaccine booster-elicited serum, but evades most convalescent serum and therapeutic antibodies

Sabrina Lusvarghi *et al.*

Corresponding author: Carol D. Weiss, carol.weiss@fda.hhs.gov

DOI: 10.1126/scitranslmed.abn8543

**The PDF file includes:**

Tables S1 and S2

**Other Supplementary Material for this manuscript includes the following:**

MDAR Reproducibility Checklist

## Supplementary Materials

**Table S1. Genotypes of severe acute respiratory syndrome coronavirus 2 (SARS-CoV-2) isolated from infected individuals.**

| Sample # | Pangolin 3.1.17 (2021-12-06) | Spike Substitutions | Spike Deletions | Accession |
|---|---|---|---|---|
| **Conv-01** | AY.74 | T19R, G142D, R158G, A222V, L452R, T478K, D614G, P681R, D950N | E156-, F157- | OM000262 |
| **Conv-02** | AY.25 | T19R, S112L, G142D, R158G, L452R, T478K, D614G, P681R, D950N | E156-, F157- | OM000263 |
| **Conv-03** | AY.47 | T19R, G142D, R158G, A222V, V289I, L452R, T478K, D614G, P681R, D950N | E156-, F157- | OM000264 |
| **Conv-04** | B.1.617.2 | T19R, K77T, G142D, R158G, G181V, L452R, T478K, D614G, A653V, P681R, D950N | E156-, F157- | OM000265 |
| **Conv-05** | B.1.617.2 | T19R, K77T, G142D, R158G, G181V, L452R, T478K, D614G, A653V, P681R, D950N | E156-, F157- | OM311576 |
| **Conv-06** | AY.14 | T19R, G142D, R158G, L452R, T478K, D614G, P681R, D950N | E156-, F157- | OM000266 |
| **Conv-07** | AY.14 | T19R, G142D, R158G, L452R, T478K, D614G, P681R, D950N | E156-, F157- | OM000267 |
| **Conv-08** | AY.25 | T19R, S112L, G142D, R158G, L452R, T478K, D614G, P681R, D950N | E156-, F157- | OM000268 |
| **Conv-09** | B.1.617.2 | T19R, T95I, G142D, R158G, L452R, T478K, D614G, P681R, D950N, G1124V | E156-, F157- | OM000269 |
| **Conv-10** | AY.62 | T19R, G142D, R158G, A222V, L452R, T478K, D614G, P681R, G946V, D950N | E156-, F157- | OM000270 |
| **Conv-11** | AY.25 | T19R, G142D, R158G, L452R, T478K, D614G, P681R, D950N | E156-, F157- | OM000271 |
| **Conv-12** | AY.44 | T19R, T22I, G142D, R158G, L452R, T478K, D614G, P681R, D950N | E156-, F157- | OM000272 |
| **Conv-13** | AY.119 | T19R, T95I, G142D, R158G, L452R, T478K, D614G, P681R, D950N | E156-, F157- | OM000273 |
| **Conv-14** | B.1.617.2 | T19R, G142D, R158G, L452R, T478K, D614G, P681R, D950N | E156-, F157- | OM000274 |
| **Conv-16** | B.1.617.2 | T19R, T95I, G142D, R158G, L452R, T478K, D614G, P681R, D950N | E156-, F157- | OM000276 |
| **Conv-18*** | B.1.351 | | | |
| **Conv-19** | B.1.351 | D80A, D215G, K417N, E484K, N501Y, D614G, A701V | | OM000278 |
| **Conv-20** | B.1 | D614G | | OM000279 |
| **Conv-21** | B.1 | D614G | | OM000280 |

| | | | | |
|---|---|---|---|---|
| **Conv-22** | B.1 | D614G | | OM000281 |
| **Conv-23** | B.1 | D614G | | OM000282 |
| **Conv-24** | B.1 | D614G | | OM000283 |
| **Conv-25** | B.1 | D614G | | OM000284 |
| **Conv-26** | B.1 | D614G | | OM000285 |
| **Conv-27** | B.1 | D614G | | OM000286 |
| **Conv-28** | B.1 | D614G | | OM000287 |
| **Conv-29** | B.1 | D614G | | OM000288 |
| **Conv-30** | B.1.1.7 | N501Y, A570D, D614G, P681H, T716I, S982A, D1118H | H69-, V70-, Y144- | OM000289 |
| **Conv-31** | B.1.1.7 | N501Y, A570D, D614G, P681H, T716I, S982A, D1118H | H69-, V70-, Y144- | OM000290 |
| **Conv-32** | B.1.1.7 | N501Y, A570D, D614G, P681H, T716I, S982A, D1118H, K1191N | H69-, V70-, Y144- | OM000291 |
| **Conv-33** | B.1.1.7 | N501Y, A570D, D614G, P681H, T716I, S982A, D1118H | H69-, V70-, Y144- | OM000292 |
| **Conv-35** | B.1.2 | D614G | | OM000294 |
| **Conv-36** | B.1.2 | G257D, D614G | | OM000295 |
| **Conv-37** | B.1.2 | D614G | | OM000296 |
| **Conv-38** | B.1.2 | D614G | | OM000297 |
| **Conv-39** | B.1.2 | D614G | | OM000298 |
| **Conv-40** | B.1.2 | D614G | | OM000299 |

*The SARS-CoV-2 sequence information from this individual was not available.

**Table S2. $NT_{50}$ values for each convalescent serum sample against D614G, Delta, and Omicron.**

| | | D614G | | Delta | | Omicron | |
|---|---|---|---|---|---|---|---|
| **Sample #** | **Variant** | **Mean** | **SD** | **Mean** | **SD** | **Mean*** | **SD** |
| **Conv-20** | **B.1** | 1626.5 | 213.7 | 6135.0 | 654.8 | 259.4 | 16.0 |
| **Conv-21** | **B.1** | 2333.3 | 355.9 | 1717.5 | 682.4 | 20.0 | |
| **Conv-22** | **B.1** | 392.4 | 67.3 | 267.0 | 58.4 | 20.0 | |
| **Conv-23** | **B.1** | 1121.4 | 415.9 | 95.1 | 32.5 | 20.0 | |
| **Conv-24** | **B.1** | 177.1 | 36.6 | 174.6 | 26.4 | 20.0 | |
| **Conv-25** | **B.1** | 2781.2 | 885.0 | 3068.0 | 502.0 | 72.0 | 14.6 |
| **Conv-26** | **B.1** | 2116.5 | 473.0 | 2602.5 | 415.1 | 20.0 | |
| **Conv-27** | **B.1** | 80.5 | 24.2 | 34.5 | 20.5 | 20.0 | |
| **Conv-28** | **B.1** | 1849.8 | 320.6 | 122.3 | 16.2 | 20.0 | |
| **Conv-29** | **B.1** | 92.2 | 28.5 | 20.0 | | 20.0 | |
| **Conv-35** | **B.1.2** | 393.9 | 92.2 | 1384.2 | 523.5 | 20.0 | |
| **Conv-36** | **B.1.2** | 3375.0 | 807.0 | 1890.0 | 8.5 | 20.0 | |
| **Conv-37** | **B.1.2** | 1012.5 | 137.6 | 675.1 | 199.2 | 20.0 | |
| **Conv-38** | **B.1.2** | 1484.3 | 145.3 | 399.1 | 28.2 | 20.0 | |
| **Conv-39** | **B.1.2** | 427.0 | 68.3 | 458.7 | 143.0 | 20.0 | |
| **Conv-40** | **B.1.2** | 226.4 | 36.9 | 452.1 | 77.6 | 20.0 | |
| **Conv-30** | **B.1.1.7** | 1191.3 | 168.4 | 472.2 | 206.0 | 20.0 | |
| **Conv-31** | **B.1.1.7** | 37112.2 | 8284.0 | 13937.5 | 2715.9 | 100.0 | 17.9 |
| **Conv-32** | **B.1.1.7** | 10178.0 | 2532.0 | 6054.5 | 1967.4 | 20.0 | |
| **Conv-33** | **B.1.1.7** | 369.6 | 89.6 | 412.9 | 174.1 | 20.0 | |
| **Conv-18** | **B.1.351** | 166.2 | 33.7 | 94.1 | 22.6 | 61.3 | 15.6 |
| **Conv-19** | **B.1.351** | 1110.5 | 249.3 | 852.7 | 242.0 | 189.2 | 31.7 |
| **Conv-4** | **B.1.617.2** | 42.0 | 2.4 | 130.4 | 4.5 | 20.0 | |
| **Conv-5** | **B.1.617.2** | 610.0 | 76.4 | 1389.5 | 364.2 | 65.7 | 6.7 |
| **Conv-9** | **B.1.617.2** | 176.0 | 7.3 | 2217.0 | 165.5 | 57.6 | 10.8 |
| **Conv-14** | **B.1.617.2** | 1250.0 | 35.4 | 64680.5 | 2305.9 | 381.7 | 55.0 |
| **Conv-16** | **B.1.617.2** | 20533.7 | 4313.7 | 14482.0 | 1968.6 | 224.1 | 28.0 |
| **Conv-1** | **AY.74** | 322.6 | 44.5 | 8303.0 | 2190.6 | 61.2 | 19.4 |
| **Conv-2** | **AY.25** | 2368.5 | 67.2 | 58860.0 | 19859.8 | 1713.0 | 217.8 |
| **Conv-3** | **AY.47** | 8832.5 | 1451.7 | 44544.5 | 9013.5 | 299.8 | 29.6 |
| **Conv-6** | **AY.14** | 303.4 | 44.4 | 13762.5 | 1501.2 | 31.2 | 15.9 |
| **Conv-7** | **AY.14** | 129.2 | 29.4 | 640.5 | 30.5 | 20.0 | |
| **Conv-8** | **AY.25** | 1195.0 | 15.6 | 7733.0 | 553.0 | 126.4 | 5.7 |

| | | | | | | | |
|---|---|---|---|---|---|---|---|
| **Conv-10** | **AY.62** | 1594.0 | 202.2 | 31861.0 | 6280.5 | 81.5 | 8.8 |
| **Conv-11** | **AY.25** | 160.6 | 2.6 | 1950.5 | 299.1 | 72.1 | 14.5 |
| **Conv-12** | **AY.44** | 2520.0 | 383.3 | 24904.5 | 4666.2 | 1921.0 | 1189.4 |
| **Conv-13** | **AY.119** | 1260.0 | 164.0 | 22082.5 | 6200.6 | 344.0 | 61.3 |

*Titers below the lowest dilution tested (1:40 dilution) were set as 20. $NT_{50}$ = 50% neutralization titer. SD standard deviation.
